# Living Microneedles for Intradermal Delivery of Beneficial Bacteria

**DOI:** 10.1021/acsbiomaterials.4c02230

**Published:** 2025-01-20

**Authors:** Caroline Hali Alperovitz, Noa Ben David, Yuval Ramot, Adi Gross, Boaz Mizrahi

**Affiliations:** † Faculty of Biotechnology and Food Engineering, Technion—Israel Institute of Technology, Haifa 3200003, Israel; ‡ Department of Dermatology and Venereology, Hadassah−Hebrew University Medical Center, Jerusalem 9112001, Israel

**Keywords:** microneedles, PVA, PVP, *Bacillus subtilis*, skin, bacterial infections

## Abstract

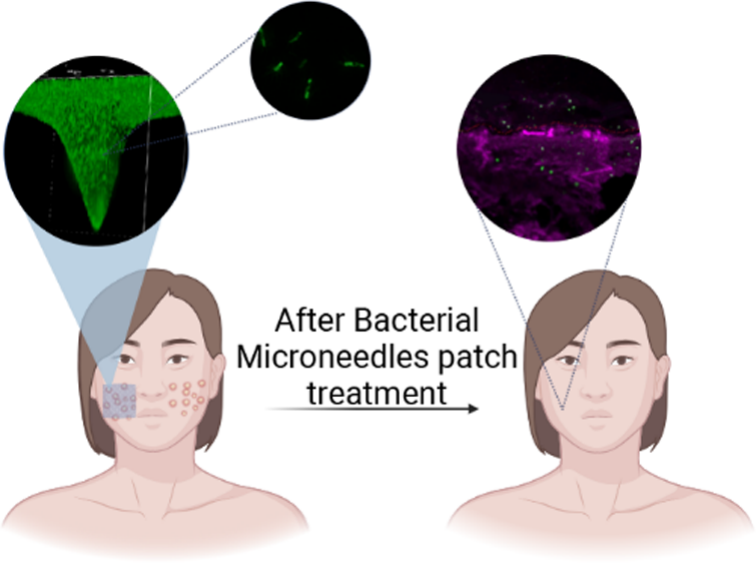

The skin, our first line of defense against external threats, combines a physical barrier and a rich microbial community. Disruptions of this community, for example, due to infectious injury, have been linked to a decrease in bacteria diversity and to mild to severe pathological conditions. Although some progress has been made in the field, possibilities/procedures for restoring the skin microbiome are still far from ideal. The objective of this study was to design and evaluate a dissolvable poly(vinyl alcohol)/polyvinylpyrrolidone microneedle (MN) patch containing live *Bacillus subtilis*. According to the plan, bacteria were distributed equally throughout the patch without compromising the morphology and mechanical properties of the needles. *B. subtilis* was successfully released from the MNs, reaching a logarithmic growth phase after 5 h. These MNs demonstrated remarkable antibacterial activity against the Gram-positive pathogenic *S. pyogenes*, *S. aureus*, and *C. acnes*, while the empty control MNs showed no such activity. Finally, mice were inserted with a single MN patch loaded with GFP-*B. subtilis* presented significantly higher total radiance efficiency (TRE) values compared to the empty-MN mice throughout the entire experiment. This concept of incorporating live, secreting bacteria within a supportive MN patch shows great promise as a bacterial delivery system, offering a potential shift from conventional pharmacological approaches to more sustainable and symbiotic therapies.

## 1 Introduction

The skin, our largest organ, serves as a barrier against external threats while hosting a wide range of various microorganisms.^1^ Among these, bacteria play a crucial role in shaping and maintaining skin health and function.^2^ Recent skin microbiome studies have revealed the multifaceted link between skin health and the microbial community inhabiting it, leading to new therapeutic approaches based on beneficial bacteria.^3−5^ For example, VITSAMJ1, a Gram-positive lactobacilli, enhanced wound healing in rats,^6^ while topical administration of *Streptococcus thermophilus* improved atopic dermatitis symptoms in humans.^7^*Bacillus subtilis* (*B. subtilis*) is a probiotic bacterium capable of producing antimicrobial peptides that inhibit the growth of pathogenic bacteria.^8−10^ The positive role of *B. subtilis* in treating inflammatory skin conditions has been demonstrated in several diseases, including acne, impetigo, and cellulitis.^11,12^ Since *B. subtilis* exhibits resistance to environmental stresses, it can potentially be used in skincare formulations without multifaceted pretreatments.^13,14^ Although bacterial delivery may offer many benefits, conventional skin delivery systems, such as creams and gels, are far from ideal. Such formulations are often toxic to the bacteria, have limited penetration depth and inefficient targeting, and in some cases are accompanied by serious adverse effects caused by the active agent.^15−17^

Dissolvable microneedles (MNs) are a minimally invasive apparatus composed of micrometer-sized needles arranged on a small soluble patch. Dissolvable MNs can painlessly bypass the skin’s epidermis and release their content directly into the inner skin layers.^18^ Several bacterial delivery systems based on MNs have been proposed. These studies and applications include dissolvable MNs containing *lactobacillus* bacteria that safely penetrate the skin while producing lactic acid;^19^*B. subtilis* MNs that demonstrated comparable antifungal effects as demonstrated by Ketoconazole;^20^ and pneumatic MN patches containing live *Enterobacter aerogenes* that actively enhance drug release.^21^ However, while the effectiveness of these systems has been proven, their long-term stability, proliferation capabilities, and their efficacy against Gram-positive pathogens are still unknown.^22^

Here, we hypothesized that dissolvable MNs incorporated with *B. subtilis* will overcome some of the shortcomings of the currently used delivery systems for the intradermal delivery of beneficial bacteria. Specifically, we present dissolvable MNs made of poly(vinyl alcohol) (PVA) and polyvinylpyridine (PVP) that are incorporated with *B. subtilis*. By encapsulating probiotic bacteria within dissolvable carriers, these MNs may enable minimally invasive and painless administration while ensuring the viability and stability of the bacteria. We chose PVA and PVP (1:2 w/w) to serve as the main components of the MNs due to their hydrophilic nature and low cytotoxicity.^23,24^ In previous studies, we have shown that MN of similar composition is biocompatible without cytotoxic effects under in vitro and in vivo conditions.^25^ Furthermore, these polymers possess excellent mechanical strength, which is crucial for maintaining structural integrity during skin penetration.^25,26^ Here, we describe the manufacturing process and optimization of the MNs, study their morphology, mechanical properties, and bacterial viability, and report on the in vitro and in vivo performance of the system.

## 2 Materials and Methods

### 2.1 Materials

PVA (*M*_w_ = 13,000–23,000 g·mol^–1^, 99% hydrolysis), PVP, K90 (*M*_w_ = 360000 g·mol-1), and chloramphenicol (Sigma Chemicals, St. Louis, MO, USA). O.C.T (Scigen, CA, USA). Bacto tryptone, bacto yeast extract, bacto agar, and tryptic soy agar (TSA) (Becton Dickenson, NJ, USA), NaCl (Bio-Lab Chemicals, Jerusalem), MgSO_4_ (Spectrum Chemical, CA, USA), KH_2_PO_4_ (Riedel-de Haën, Munich). Ultrapure water was obtained (Bio-Lab, Israel). *B. subtilis* 3610 was provided by Prof. Ilana Kolodkin-Gal’s lab,^27^ Weizmann Institute of Science, Israel. Bacterial cultures were American Type Culture Collection (ATCC) strains: *Staphylococcus aureus* 25923 (*S. aureus*), *Streptococcus pyogenes* 19615 (*S. pyogenes*), and *Cutibacterium acnes* 6919 (*C. acnes*).

### 2.2 MN Fabrication and Characterization

#### 2.2.1 Fabrication

*B. subtilis* were incubated in Luria–Bertani (LB) medium at 37 °C while shaken at 200 rpm until turbidity reached 1 OD at 600 nm. The resulting suspension was then centrifuged at 4000 rpm for 5 min, the supernatant was discarded, and the pellet was obtained. PVA/PVP MNs were fabricated using the micromolding technique.^28^ In brief, aqueous solutions containing PVA and PVP (10 wt % each) were combined to achieve a final volume ratio of 1:2 PVA/PVP. *B. subtilis* pellet (derived from 1 mL suspensions, as described above) were added to polymer solutions (1 mL) and 110 μL of the bacterial/polymers suspension were applied onto a PDMS mold (8 mm × 8 mm area, with a cavity height of 500 μm and a base diameter of 200 μm; Micropoint Technologies Pte, Ltd. Singapore). Molds were spun at 4000 rpm for 1 min (Laurell model WS-650MZ-23NPPB, North Wales, USA), followed by a second casting (110 μL) of the same solution and another spin cycle. After being dried for 24 h at room temperature, the MNs were gently removed from the molds using tweezers and stored in a desiccator until use. Control MNs of the same composition but lacking *B. subtilis* were produced and stored under similar conditions.

#### 2.2.2 Morphology

The morphology of MNs containing *B. subtilis* was observed using a scanning electron microscope (FEI E-SEM Quanta 200, Eindhoven, The Netherlands) and a ProX tabletop scanning electron microscope (Phenom-World BV, Eindhoven, The Netherlands).

### 2.3 Mechanical Strength

The mechanical strength of *B. subtilis* MNs with various bacterial loads was assessed by a compression test^29^ using a Lloyd TA1 Texture Analyzer (Lloyd Instruments Ltd., West Sussex, UK) equipped with a 10 N load cell. MNs were fabricated using varying ratios of *B. subtilis* suspension. PVA/PVP solutions (1 mL) were mixed with a bacterial pellet derived from a 2 mL suspension to achieve a polymer/bacteria ratio of 1:2 (v/v). Ratios of 1:1 and 1:0.5 were also attained using pellets from 1 and 0.5 mL of bacterial starters, respectively. The MNs were then attached, face up, to the center of the bottom plate (11.5 cm in diameter) by using a carbon patch. Compression was applied by the upper plate at a rate of 1 mm/min and was limited to 330 μm after reaching the contact point with the MNs. The compression force was monitored continuously and expressed as N/needle. Compression results were compared with those for MNs without bacteria (*n* = 4).

### 2.4 Thermogravimetric Analysis (TGA)

MNs were prepared and dried at room temperature and stored overnight in a desiccator. Water content was then examined using TGA (TGA5500, New Castle, USA). Empty and bacterial MNs were placed in crucibles (*n* = 6). Weight changes were measured from 27 to 200 °C under a flowing nitrogen atmosphere at a heating rate of 20 °C/min. Results are presented as weight loss (%) over temperature and weight derivative (d*W*/d*t*).

### 2.5 *B. subtilis* Release

The impact of the MNs’ drying conditions on the survival of *B. subtilis* was evaluated by examining the growth patterns of bacteria released from MNs following desiccator or room temperature drying. MNs with *B. subtilis* and free bacteria were cultured at 37 °C, and their growth was monitored for 24 h using a spectrophotometer at λ = 600 nm (Synergy H1 Plate Reader, Biotech Instruments Inc., Winooski, VT, USA).

### 2.6 Bacterial MNs Stability

For stability assessments, bacterial MNs were prepared and stored at monitored humidity for 3 and 6 months. Then, MNs were cut into quarters and grown in a 24-well plate containing 1.5 mL of LB medium in each well. Growth kinetics were documented over 24 h at 37 °C and monitored using a spectrophotometer (*n* = 4).

### 2.7 Loading and Release of Bacteria on/from MNs

#### 2.7.1 Transformation of *B. subtilis*

The release of *B. subtilis* from the MNs and its proliferation were assessed using GFP-labeled bacteria. First, we performed a *transformation* with the PAD 43–25 plasmid.^30^*Escherichia coli* (*E. coli*) competent cells were prepared through electroporation using a Gene Zapper (IBI, New Haven, CT) (2 mm cuvette, 2500 V, 400 Ω, 21 μF). The PAD 43–25 plasmid was isolated from *E. coli* using a plasmid purification kit (Promega, MDN, USA) according to the manufacturer’s instructions. Then, a single colony of *B. subtilis* was cultured in 5 μg/mL MC medium^31^ at 37 °C and 200 rpm. Cells were harvested at mid log phase by centrifugation at 4000 rpm for 5 min at room temperature and suspended to achieve a final OD_600_ of 0.5. For the transformation, 2 μg of isolated plasmid DNA was added to 300 μL of *B. subtilis* suspension and incubated at 37 °C for 3 h with shaking at 200 rpm. Transformed cells were then plated onto Luria Agar (LA) plates supplemented with chloramphenicol and incubated at 37 °C overnight.

#### 2.7.2 Bacteria Distribution in MNs

The dispersion profile of the bacteria in the MNs was determined by using confocal microscopy. MNs containing *B. subtilis* expressing the pAD43-25 plasmid for constitutive green fluorescent protein (GFP) were positioned between a glass slide and a cover slide with 1% agarose gel. Confocal images were captured at 2 μm intervals on a spinning disc confocal (Nikon, Japan). GFP entrapment by *B. subtilis* was verified using a super-resolution microscope (Lattice SIM/STED/STORM, Zeiss, DE).

#### 2.7.3 Bacterial Penetration In Vitro

The release of *B. subtilis* from the MNs and its penetration were examined using a Cytation 5 imager equipped with an automated digital microscope (Cytation 5, Agilent, CA US). GFP-labeled *B. subtilis* MNs were fabricated as described in Section 2.2. These MNs were then fixed at the center of a 6-well plate filled with LA, and growth was monitored over 24 h. As a way of comparison, an empty MN patch was also observed under similar conditions.

### 2.8 Antibacterial Activity of *B. subtilis* MNs

The antibacterial capabilities of the *B. subtilis* MNs were assessed against *C. acnes*, *S. aureus*, and *S. pyogenes* using the disk diffusion assay. *S. aureus* and *S. pyogenes* were seeded (100 μL of 10^8^ CFU/ml) on LA plates using a sterile Drigalski spatula. *B. subtilis* MNs were cut into circles (6 mm) and positioned on each plate using a sterile spatula. Plates were incubated at 37 °C for 48 h under aerobic conditions, and the diameter of the inhibition zone was determined. The efficiency against *C. acnes*, which was cultured on both TSA and TSA-blood agar at 37 °C for 24 h, was determined under anaerobic conditions (Ar-filled glovebox, O_2_ < 0.2 ppm). For comparison, the efficacy of empty MNs and of free *B. subtilis* were evaluated using the same procedures. A *B. subtilis*-MNs patch (I), a disc infused with 5 μL *B. subtilis* starter (II), and an empty MN patch (III) were placed on each plate. Inhibition zones were measured using ImageJ and normalized by the initial area of each tested patch. To identify and validate the inhibiting bacteria, each sample of the suspected *B. subtilis* colonies was analyzed using the BBL Crystal kit (Gram-Positive ID Kit-BD, Maryland, USA), according to manufacturer’s instructions.

### 2.9 Bacterial Colonization

Six 8-week-old female Balb/C nude mice were purchased from Envigo (Jerusalem, Israel) and maintained under SPF conditions and 12/12 h light/dark cycles. The study was carried out according to the Animal Welfare (Animal Protection) Law (1994) and approved by the Council for Experiments on Animal Subjects at the Israeli Ministry of Health (No. IL0910523H). Mice were divided randomly into two groups: (1) Mice that were administered with GFP-*B. Subtilis* MNs and (2) mice that were administered with empty PVA/PVP MNs (*n* = 3 for each group). Mice were anesthetized with 1% isoflurane (Piramal Critical Care, Inc. PA, USA) in oxygen, and square MN patches (64 mm^2^) were placed at the center of each mouse’s dorsal region. Finally, 5 μL of ultrapure water (Bio-Lab, Israel) was applied to each patch for better fixation. The fluorescent signal expressed by the bacteria was determined by the TRE of each region of interest (ROI) as measured by an in vivo imaging system (IVIS) in the 500–540 nm wavelength range over 9 h. To validate the intradermal delivery of bacteria in Balb/C nude mice, empty and *B. subtilis*-MNs were inserted into the dorsal region, and representative tissues were collected. These samples were frozen in an OCT (24 h, −80 °C) and sliced to a thickness of 14 μm using a cryostat (Leica, Wetzlar, Germany). The sliced tissue samples were studied using a spectral microscope (LSM 880—pruight confocal with MP laser, Zeiss, DE) to differentiate between the bacterial GFP signal and tissue background noise.

### 2.10 Statistics

Statistical analyses of the characterization, antibacterial activity, and in vivo bacterial load results were conducted using Prism 5 software (GraphPad, CA). The significance between groups was determined using a *t-*test, with *p*-values <0.01 and *n* = 4 and *n* = 3 for in vitro and in vivo characterizations, respectively.

## 3 Results and Discussion

### 3.1 Fabrication and Morphology

**Figure 1:**
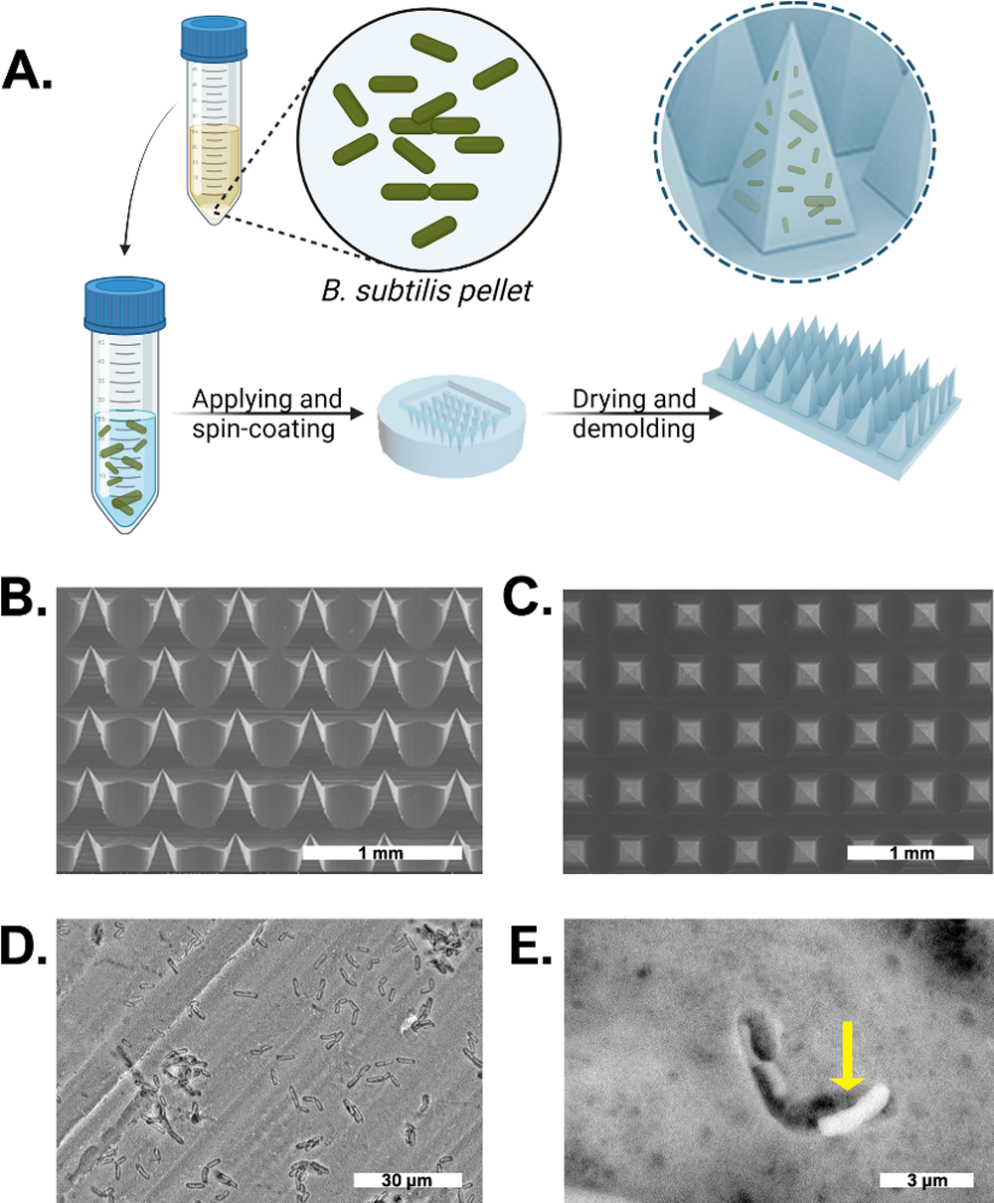
Fabrication of bacterial MNs and their morphology. (A) Schematic illustration of the fabrication of *B. subtilis* MNs. (B) Side view SEM images of MNs containing *B. subtilis*. (C) Top view of the same patch. (D) Surface view SEM images of MNs containing *B. subtilis*. (E) Single *B. subtilis* as observed on the surface using SEM (bacterium is indicated by a yellow arrow).

*B. subtilis* MNs were fabricated by mixing aqueous PVA/PVP (1:2 v/v) solution with *B. subtilis* pellet (derived from 1 mL of starter at 1 OD, 600 nm). The bacterial suspension was applied onto a PDMS mold, spun, and left to dry for 24 h at room temperature (Figure 1A). Empty MNs with identical polymeric compositions but without bacteria were fabricated using the same technique. *B. subtilis* MNs showed a defined structure that was characterized by a pyramidal morphology and an aligned structure with regular spaces of approximately 490 μm between each two bases, as dictated by the mold (Figure 1B,C). The presence of *B. subtilis* did not affect the formation of arrays, as was evident from the SEM images. A distinctive distribution of the bacteria was seen on the surface of the needles (Figure 1D), with the bacteria maintaining its typical rod-like shape (Figure 1E). Thus, the fabrication of MNs using PVA and PVP enables structural uniformity of the final patch constructions.^32,33^ The number of live bacteria in the MNs was assessed by dissolving a single MN patch onto LA plates (*n* = 4), followed by incubation and colony counting. Each patch contained around 1 × 10^5^ CFU, 2-fold less than the initial bacterial count in the bacterial/polymer suspension. The decrease in bacterial count can be explained by the drying process and nutritional stress during MN fabrication. The loss of water results in mechanical stress on the cell membrane and a possible membrane phase transition to a gel, both of which may result in cell death.^34^ We note that the results of recent clinical trials demonstrate that the use of lactobacilli at dosages ranging from 10^5^ to 10^10^ CFU is an efficient and cost-effective way to treat chronic wounds and improve healing processes.^35^

### 3.2 Mechanical Behavior

**Figure 2:**
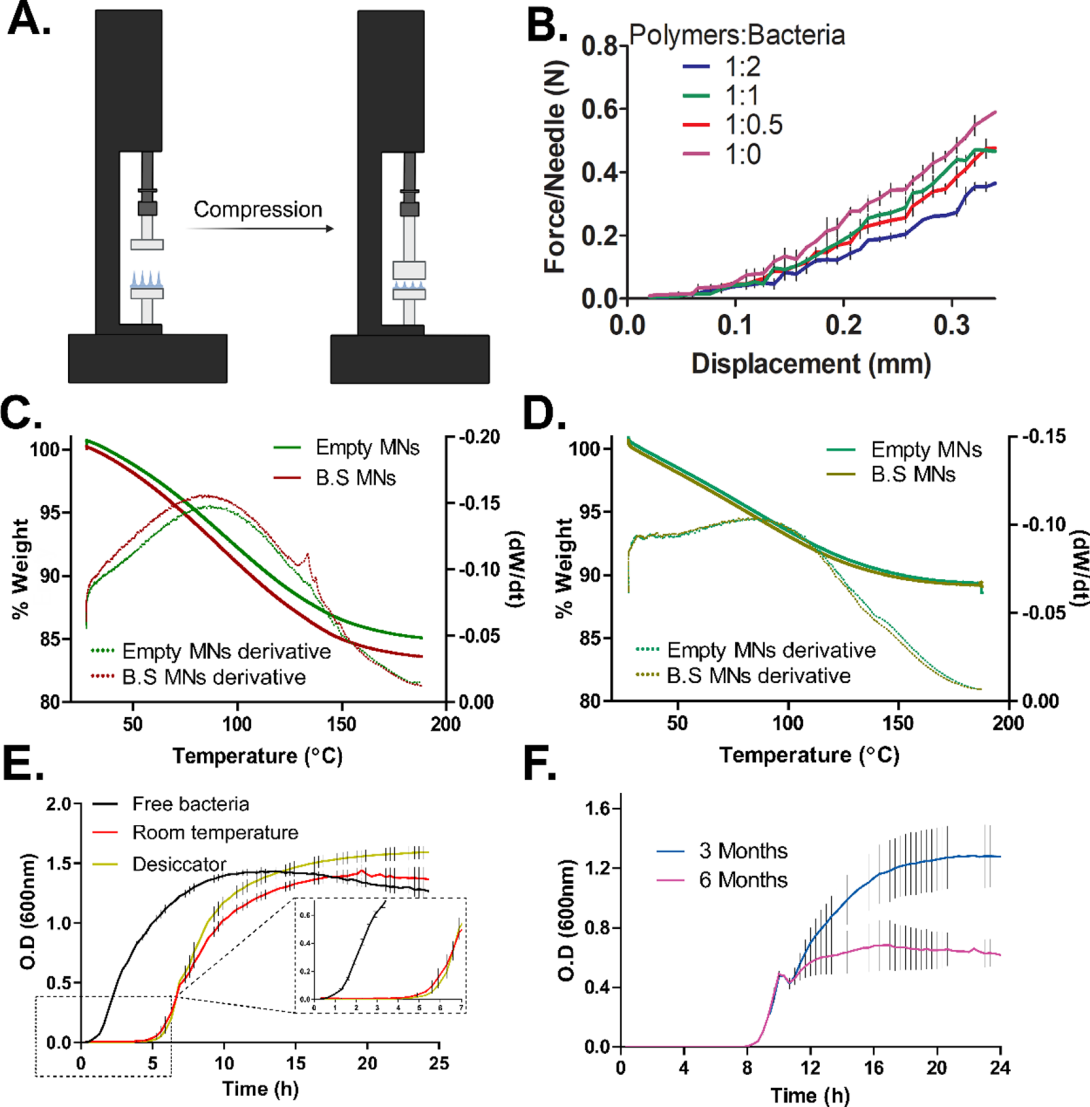
Preparation conditions and characterization of *B. subtilis*-MNs. (A) Tensiometer machine used for compression test. (B) Compression test for various bacteria/polymers ratios. (C) Thermogravimetric curves of MN samples dried at room temperature. (D) Thermogravimetric curves of MN samples were dried in a desiccator. Dashed lines represent the derivative of the weights (d*W*/d*t*). (E) Growth dynamics for free *B. subtilis* and from MNs. (F) Recovery of *B. subtilis* from MN after 3 and 6 months.

To function as an effective living system, MNs must enable insertion and passage through the skin layers while containing *B. subtilis*. We chose to mix PVA and PVP aqueous solutions at a 1:2 volume ratio based on our experience with MN manufacturing.^25^ This composition was found to be optimal in terms of mechanical strength, MNs’ morphology, and stability. The mechanical strength of PVA/PVP MNs, fabricated from varying ratios of polymeric solution to *B. subtilis* suspension (V/V), was assessed by compression tests (Figure 2A). Force applied on the needles was monitored and presented as N/needle (Figure 2B). Increasing the bacterial load decreased the mechanical strength of the needles. For example, the 1:2 (polymer/bacteria) ratio showed the lowest mechanical strength, while the 1:0.5 had the highest mechanical strength. MNs without bacteria exhibited the highest force-to-needle ratio throughout the experiment. This negative effect on the mechanical properties of the needles is probably due to the plasticizing and softening effect of bacteria, mostly through moisture retention.^36,37^ Another explanation for the softening effect of the bacteria can be the interruption with polymers' intermolecular forces which disorganizes the structure.^38^ It is unlikely that *B. subtilis* plays a significant role in the degradation of the dried MNs, as degradation of PVA and PVP by *B. subtilis* 3610 has not been documented,^39^ since degradation of petroleum compounds by bacteria generally requires higher temperatures (>60 °C) and since the bacteria in the dry MNs are in a dormant stage, where they are inactive.^40^

Nevertheless, the travel curves of the tested MNs showed a smooth and continuous plot with no signs of breakage or bending (Figure 2B).^37^ Consequently, MNs with a 1:1 ratio were chosen for further examinations as they contain a high bacteria concentration along with a needle force of approximately 0.4 N, which is well within the standard force range for skin penetration.^41,42^

### 3.3 TGA

Water activity plays a significant role in the viability of beneficial bacteria and may negatively impact the overall stability and shelf life of some products as it induces the proliferation of bacteria and other microorganisms while supporting their enzymatic activity.^43^ Therefore, the moisture content of MNs fabricated at room temperature (Figure 2C) was analyzed by TGA and compared with that of MNs fabricated under vacuum desiccation (Figure 2D). Room-temperature drying led to final weights of 85.1% and 83.6% (of initial weight) for empty and bacterial MNs, respectively, which indicates a lower water content in the empty MNs. One major peak of the derivative (d*W*/d*t*, representing weight loss changes over time) occurs around 90 °C while a smaller peak occurs around 135 °C. These peaks are associated with free water and bound water, respectively.^44,45^ For PVA/PVP blends, typical weight-loss behavior within the temperature ranges of 75–135 °C is attributed to water evaporation.^46^ Thus, all weight loss in this experiment is related to water evaporation, and so it follows that *B. subtilis*-MNs had a higher initial moisture content. Focusing on the derivative’s second peak, it is noticeable that bacterial MNs exhibited a higher peak magnitude than did the empty MNs, indicating that more water molecules are bound when bacteria are present.^47^ It can therefore be concluded that *B. subtilis* facilitates water retention when combined with PVA/PVP, possibly due to the production and secretion of exopolysaccharides that effectively decrease water loss.^48^ As for desiccated MNs (Figure 2D), no significant difference was seen between the final weights of empty and bacterial MNs, which were approximately 89% of the initial weights. Accordingly, when harsher drying conditions prevailed during MN preparation, bacteria had less of an effect on the final humidity content.^49^ The same tendency was observed for the derivatives of the weights, indicating that the patches dried under vacuum conditions in a dehumidified environment contained less water than those dried at room temperature.^50^ To further investigate the effect of the humidity content resulting from the different drying processes on the system’s functionality, bacterial viability, and mechanical strength, appropriate tests were conducted.

### 3.4 *B. subtilis* Viability

To examine the effect of the fabrication process on bacterial viability, the growth rate of free *B. subtilis* and of bacterial MNs was monitored in LB media using a spectrophotometer (OD = 600 nm) (Figure 2E). For a comparative analysis, an analogous experiment was conducted with empty MNs. Free *B. subtilis* taken from a fresh colony started logarithmic growth after only 1h and reached a plateau within 8 h. *B. subtilis* in MNs, on the other hand, started logarithmic growth after 5 h and reached a plateau within 15 h. OD values (600 nm) of all tested bacteria at the plateau were between 1.4 and 1.6, with dried bacteria in the desiccator presenting the highest values (OD_600 nm_ = 1.6). Thus, it is clear that the fabrication process and the dry state of the MNs have a dramatic effect on the duration of the lag phase but have had no apparent effect on the stationary phase. This observed growth behavior of *B. subtilis* in MNs can be explained by both low metabolic activity and starvation of the dried bacteria in the MNs. The excess lag phase time is therefore required for the bacteria to adapt to the new rich environment.^51^ The higher growth performance of the desiccator-dried bacteria can be explained, at least partially, by the different process parameters of the methods. A shorter processing time and lower final moisture content are preferable for maintaining bacterial viability since they decrease water activity and minimize the chance of bacterial damage.^52^ Moreover, since absorbed water tends to act like a plasticizer, the glass transition temperature of the polymeric matrix is lowered, leading to compromised storage stability of the matrix.^53^ Nevertheless, although desiccated MNs showed little improvement in bacterial growth and viability, they were fragile and hard to operate. Therefore, MNs with a 1:1 polymeric ratio that dried at room temperature were selected for further examination in this study.

### 3.5 *B. subtilis* Recovery

Since bacterial MNs can potentially become over-the-counter/store-bought therapeutic devices, it is important to assess the effect of storage time on the bacteria’s ability to recover and grow. The bacterial recovery rate of *B. subtilis* from MNs stored in a dehumidified box for 3 and 6 months was determined by using a spectrophotometer (Figure 2F). The effect of prolonged storage on bacterial growth was clearly documented: Bacteria from both kinds of MNs reached the logarithmic growth phase after about 8 h. However, bacteria from NM stored for 3 months, reached the stationary phase after 16 h, with O.D_600 nm_ = 1.2, compared with O.D_600 nm_ = 0.6 after 12h for bacteria from MN stored for 6 months. For the sake of comparison, bacteria from freshly fabricated MNs reached the logarithmic phase after 5 h and the stationary phase after 15 h with an O.D_600 nm_ = 1.4 (Figure 2E). Similar observations of the effect of storage periods have been explained by cellular damage to the bacteria, including to cell membranes and DNA.^54^ This damage can impair the bacteria’s ability to replicate and grow efficiently, leading to a longer lag phase. In general, the recovery of probiotic bacteria is also influenced by rehydration conditions, such as medium temperature, nutrition availability, and environmental pH.^55^ Since the transition from the dry environment to the rich environment was immediate so as to mimic the use conditions of the MNs on the skin, rehydration was relatively fast. This might lead to a longer lag phase, which is necessary for bacterial adjustment in terms of enzymes and gene expression.^56^ Nevertheless, further investigation and optimization are required before commercial products based on this technology can become available.

### 3.6 Bacteria Loading and In Vitro Penetration

**Figure 3:**
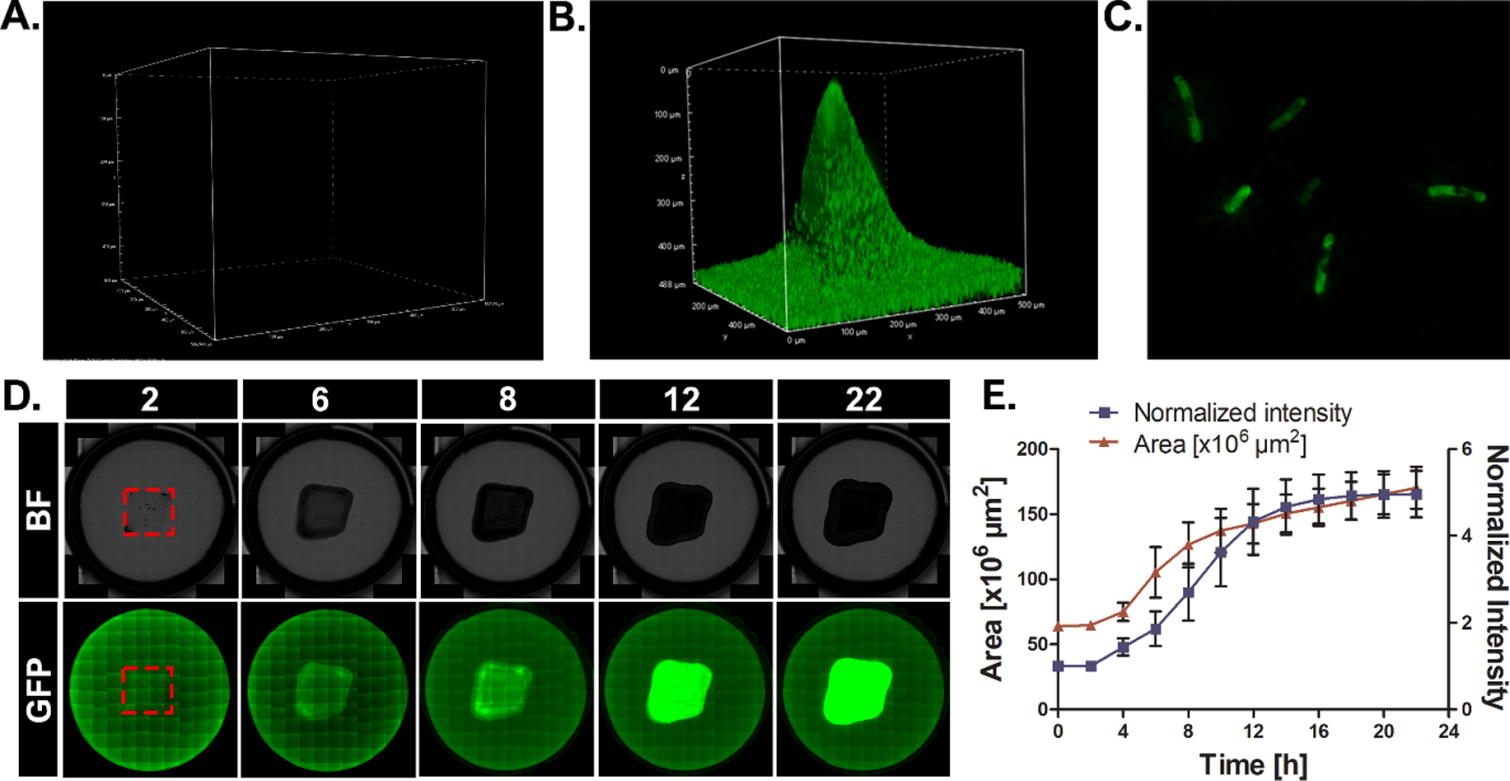
Loading and release behavior of *B. subtilis*-MNs. (A) Confocal image of Non-GFP-*B. subtilis*-MNs. (B) Confocal image depicting bacterial dispersion on the needle surface. (C) High-resolution microscopy image displaying *B. subtilis* with GFP expression within the bacteria. (D) Bacterial release from MNs: imaging of morphology under bright-field (BF) and fluorescence (GFP) microscopy (representative well). Dashed red line indicates the initial MN patch size. (E) Progression of bacterial growth in terms of area and intensity over time.

The distribution of bacteria inside the MNs and their release following patch insertion were evaluated in vitro. Confocal images of MNs with GFP-expressing bacteria were captured and compared with baseline levels of nonlabeled bacteria. While non-GFP-labeled MNs showed no indication of a fluorescence signal (Figure 3A), labeled bacteria were scattered throughout the patch (Figure 3B). High-resolution microscopy confirmed that the GFP signal was associated with the bacterial presence (Figure 3C). The ability of *B. subtilis* to penetrate a semisolid medium and colonize it was evaluated microscopically. A *B. subtilis* patch (area of 64 × 10^6^ μm^2^) was placed in a 6-well plate, and the area and intensity were monitored for 22 h using a Cytation microscope. Four hours after insertion, the intensity of bacterial GFP began increasing (Figure 3D), indicating active protein production. This increase was accompanied by a gradual growth in the bacterial area and proved to be about 2.3 times larger than the original size after 16 h (Figure 3E). Thus, our system enables the release of bacteria, their proliferation on a semisolid surface, colonization, and protein expression.

### 3.7 Antibacterial Activity of *B. subtilis*-MNs

**Figure 4:**
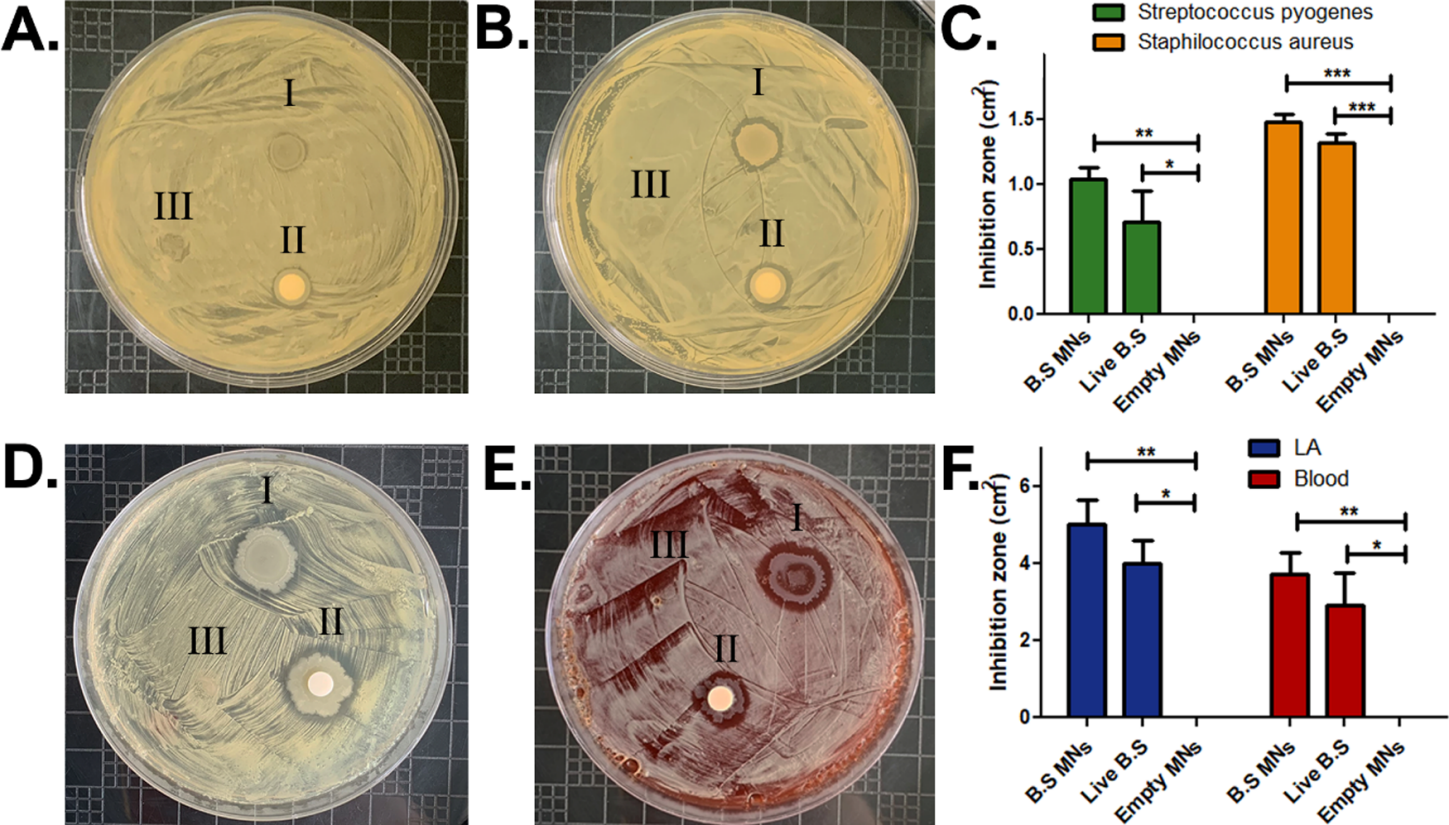
Antibacterial effect of *B. subtilis* and *B. subtilis*-MNs against *S. aureus*, *S. pyogenes*, and *C. acnes*. (A) *S. pyogenes*. (B) *S. aureus*. (C) Inhibition zones for *S. pyogenes* and *S. aureus*. (D) *C. acnes* on TSA. (E) *C. acnes* on TSB+blood agar. (F) Inhibition zones for *C. acnes*. SD of *n* = 4, *p < 0.05, **p < 0.01, ***p < 0.01.

The antibacterial efficacy of *B. subtilis*-MNs against three Gram-positive pathogenic bacteria (*S. pyogenes*, *S. aureus*, and *C. acnes*) was assessed using the Kirby–Bauer disc diffusion method.^57^ Each assay was performed on three test groups: (I) a *B. subtilis*-MNs patch; (II) a disc infused with 5 μL fresh *B. subtilis* starter; and (III) an empty MN patch. Inhibition zones were measured after incubation at 37 °C (*n* = 4). In all cases, free *B. subtilis* and *B. subtilis*-MNs demonstrated remarkable antibacterial activity compared to the empty control MNs, which showed no activity at all. Average inhibition zones for *S. pyogenes* (Figure 4A) and *S. aureus* (Figure 4B) were 1.04 and 1.47 cm^2^, respectively, while the average inhibition zones for the fresh bacteria were slightly lower compared with the MNs: 0.71 and 1.32 cm^2^ for *S. pyogenes* and *S. aureus*, respectively (Figure 4C). A similar study was conducted under anaerobic conditions on *C. acnes* (Figure 4D,E) with overnight incubation. *C. acnes* was tested on two different agar mediums: (a) in TSA medium, where the average inhibition zones for bacterial MNs and for live bacteria were 5.01 and 3.99 cm^2^, respectively, and (b) in TSA+blood medium, where the average inhibition zones were 3.70 cm^2^ for bacterial MNs and 2.92 cm^2^ for live bacteria (Figure 4F). The anaerobic assay generally mirrored the aerobic assay: bacterial MNs were able to inhibit the pathogens’ growth, with slightly better results compared with the fresh *B. subtilis*-infused disc. Here too, empty MNs exhibited no antibacterial effect, thus validating our results (Figure 4F). It may therefore be concluded that the antibacterial activity of *B. subtilis* was not affected by the fabrication processes. The strong inhibitory effect of *B. subtilis* observed here can be explained by its ability to release powerful surfactants, including surfactin, fengycin, and iturin.^9,58,59^ These biomolecules were found to disrupt the bacterial cell wall and to inhibit biofilm formation, resulting in cell death and population decrease.^60^ The pronounced antibacterial activity of *B. subtilis* under anaerobic conditions may be explained by its facultative anaerobic nature.^61,62^

Overall, *B. subtilis*-MNs showed promising results in inhibiting several types of bacteria, demonstrating that the MN fabrication process does not compromise the antibacterial activity of *B. subtilis*.

### 3.8 Bacterial Colonization

**Figure 5:**
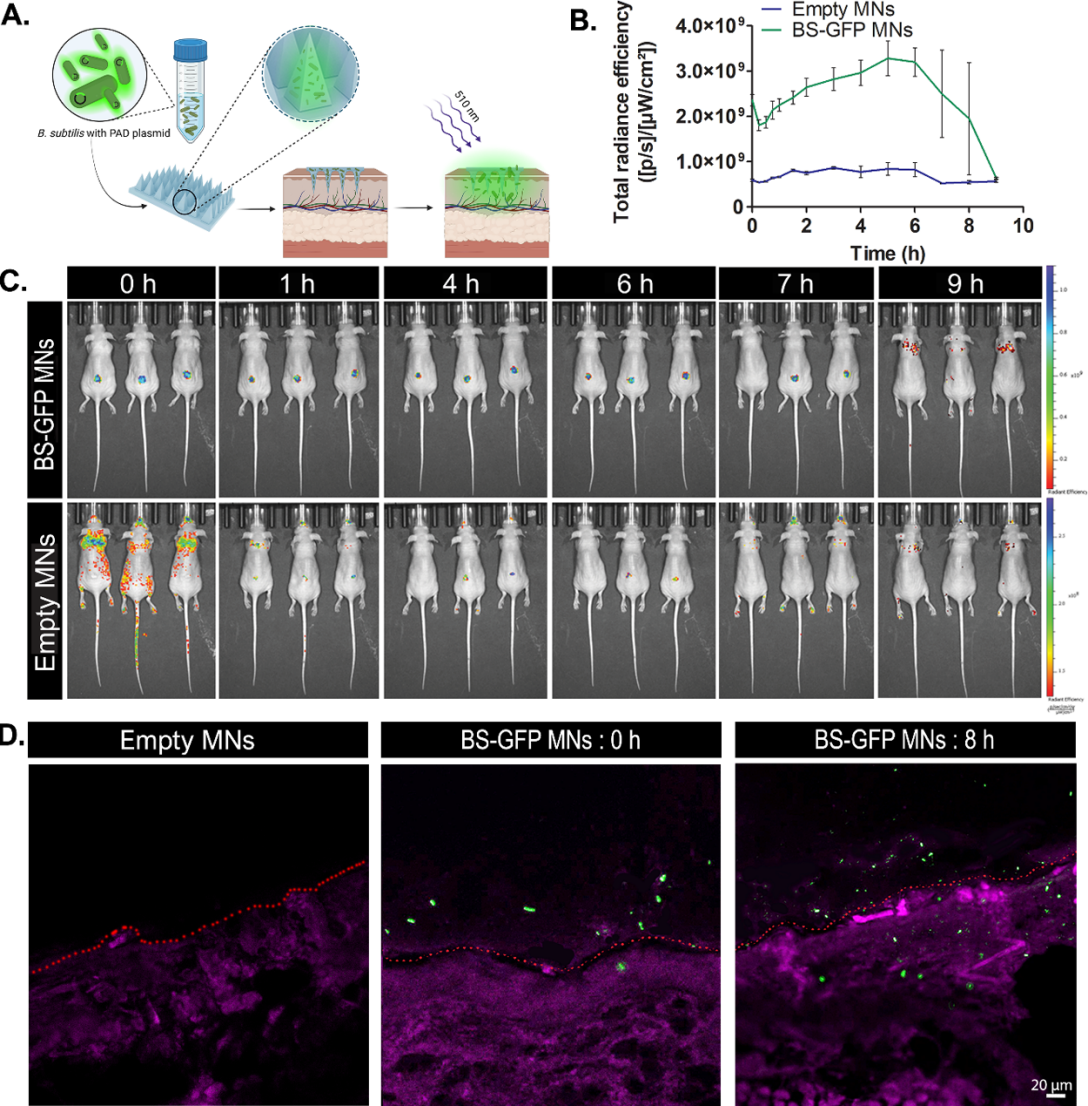
In vivo bacterial load profile. (A) Schematic illustration of the protocol. (B) Total radiance efficiency over time of tested ROIs. (C) IVIS fluorescence maps of bacterial and empty MNs at selected time points. (D) Spectral confocal microscopy image of inserted skin tissue at selected time points (*B. subtilis* are marked in green, tissue in magenta, and the tissue surface is indicated by a dashed red line).

Assessment of *B. subtilis* viability and GFP production was carried out using IVIS. The release pattern of bacteria from the patch and their GFP expression were followed. Mice were randomly divided into two groups, each containing three mice. The dorsal area of each mouse was inserted with a single patch of MNs that either contained GFP-*B. subtilis* or were empty (Figure 5A). MN patches containing GFP -B. subtilis or empty patches were prepared and the dorsal area of each mouse was inserted with a single patch. Total radiance efficiency revealed autofluorescence in both groups, but the GFP-*B. subtilis* group showed significantly higher values compared with the empty MN group throughout the entire experiment (Figure 5B). The GFP signal of the bacillus-MNs patches increased continuously from 2 × 10^9^ to 3 × 10^9^ ([ρ/s]/[μW/cm^2^]). After 1.5 h, the increase in the intensity of the signal can be attributed to the expression of newly produced GFP, mirroring the in vitro results. Five hours after administration, a gradual decrease in the TRE signal was observed. We note that IVIS has very low sensitivity to GFP placed on or inside mice skin^63,64^ and that dead cells are generally not GFP fluorescent.^65^ Therefore, this decrease in fluorescence intensity can be explained by the dissolution and breakage of the MN patch and consequently the crumbling of the matrix. The control group showed no significant changes in fluorescence signal throughout the entire experiment, supporting our explanation (Figure 5C). Since the penetration of *B. subtilis* into the skin tissue could not be verified using IVIS,^63,64^ mice with MN patches were sacrificed at selected time points. Tissues were then sliced and observed under a spectral confocal microscope to better differentiate between bacterial and tissue signals (Figure 5D). Empty MNs showed no GFP signal at any time points. At *t* = 0 h, GFP expression was observed mainly on the outer layer of the tissue, while at *t* = 8 h, bacteria reached the inner layers and were detected based on GFP expression. In addition, the ability of *B. subtilis* to become established on the skin was tested in a previous study: mice that were administrated with *B. subtilis* in Pluronic F-127 showed a shift in their microflora, which was restored 3 days post-administration.^12^ This, combined with our results, demonstrates that *B. subtilis* encapsulated in MN has the ability to penetrate the inner skin layers and potentially colonize there.

## 4 Conclusions

We developed dissolvable MNs that are incorporated with live *B. subtilis*. MNs dried at room temperature with a 1:1 PVA/PVP polymeric ratio showed the best mechanical properties compared with those of other polymeric ratios. The presence of bacteria slightly increased the water content in the needles but did not affect the formation of well-structured arrays with cells equally distributed throughout the matrix. The fabrication process of the MNs did not significantly affect the functionality of the bacteria in terms of its ability to be released from the MN and to grow and proliferate after the MN is inserted. The MNs significantly inhibit the growth of a wide range of pathogenic bacteria, an effect attributed to the powerful surfactants produced and released by *B. subtilis*. Our in vivo study showed the scattering of bacteria on both the exterior/top and inner layers of the mice’s skin. Thanks to its versatility, the system described here can easily be modified for use with other polymers or for the incorporation of health-promoting bacteria that have shown promising results, including in the restoration of a healthy skin microbiota.^66^*B. subtilis* can shape the composition of the natural flora and efficiently out-compete several human pathogenic fungi and bacteria, such as *E. coli* and *S. aureus*.^67^ In addition, *B. subtilis* exhibited anticancer and antiviral activity against influenza, herpes, and equine encephalomyelitis.^68^*Lactobacillus reuteri*, a common probiotic, having excellent antibacterial and anti-inflammatory capabilities, can be used for promoting tissue regeneration and infected wound closure;^69^ and commensal *Staphylococcus epidermidis* may contribute to skin barrier homeostasis by generating protective ceramides.^70^ Therefore, the system described here has the potential for application as a tool in dermal therapy, where intradermal injection of bacteria is useful.
